# Erectile dysfunction among hypertensive men in a rapidly developing country

**DOI:** 10.4103/0970-1591.32057

**Published:** 2007

**Authors:** Abdulbari Bener, Abdullah Al-Ansari, Mustafa Afifi, Prasad V. Krishna

**Affiliations:** Department of Medical Statistics and Epidemiology, Hamad Medical Corporation and Department Evidence for Population Health Unit, School of Epidemiology and Health Sciences, The University of Manchester, Manchester, UK; * Department of Urology, Hamad Medical Corporation, State of Qatar; ** Dept. of Non- Communicable Diseases Control, Ministry of Health (HQ), Muscat, Sultanate of Oman

**Keywords:** Epidemiology, erectile dysfunction, hypertension, International index of erectile function, Qatari's population, risk factors

## Abstract

**Background:**

Previous studies have supported the association between hypertension and erectile dysfunction (ED). In addition, a significant correlation between hypertension in men and ED has been well established.

**Objectives:**

The aim of this study was to investigate the prevalence of ED, its severity and other sexual function domains in hypertensive patients in Qatar.

**Design and Setting:**

Cross-sectional study conducted between January and December 2006 at the Hamad General Hospital.

**Subjects:**

Qatari and nonQatari nationals 25 to 75 years of age were approached to participate in this study, 642 (75.5%) gave their consent.

**Materials and Methods:**

Qatari and nonQatari men aged 25 to 75 years who gave consent were interviewed face-to-face. Sexual function was evaluated with the International index of erectile function.

**Results:**

A total of 642 (75.5%) men responded, mean age of subjects was 57.1 ± 11.9 years. The prevalence of ED in hypertensive patients was 58.3%. 21.2% of hypertensive men reported severe ED, 20.7% moderate ED and 16.4% mild ED. The comorbidities and risk factors were significantly more prominent in ED patients than in those with normal ED for hypercholesterolemia (*P*=0.009), diabetes (*P*=0.022) for hypertriglyceridemia (*P*=0.033) and myocardial ischemia (*P*=0.042).

**Conclusions:**

Our results have shown a greater prevalence of ED in the hypertensive men in the population of Qatar. The presence of another risk factor in addition to elevated blood pressure can increase the likelihood of ED.

The State of Qatar has witnessed a rapid change in many aspects of life during the last two decades. The discovery of oil in the mid-twentieth century has contributed to significant social change and Qatar, along with the other Gulf cooperation council (GCC) has experienced a rapid transition in its socioeconomic status. People in Qatar now enjoy a high standard of living; a substantial improvement over what had prevailed earlier. There has been a dramatic rise in the national economy expressed in terms of per capita income. In 2004, the gross domestic product of Qatar was higher than the rest of the GCC countries and estimated at US$ 28,270.[1] The dramatic socio-economic changes have had a great impact on urbanization and lifestyle of the Qatari community; and as a result, hypertension has become the main public health problem.[2–3]

Erectile dysfunction (ED) is a very common condition among middle-aged men.[4–16] The Massachusetts Male Aging Study[6] has shown that in men aged 40 to 70 years, the prevalence of ED is 52%. A significant association between hypertension and ED is also reported by several authors.[8–16] Hypertension is the most common comorbidity in patients with ED.[5,7] Hypertension, a major risk factor of ED, affects 29.4 million men in the United States[7] whereas in the Qatari men the prevalence rate is 32.6%.[3] This condition affects the quality of life of the patients and their wives or partners.[10] Sexual dysfunction is a frequently encountered problem in patients with hypertension and may occur as a side-effect of some types of antihypertensive medications.[8,10,13,14] Different groups of antihypertensive medications can lower blood pressure similarly, but have dissimilar effects on ED.[10] Lundberg and Biriell[13] and Doumas *et al* [14] reported that ED is more likely to be seen with alpha-blocking or alpha/beta-blocking agents and guanidine derivatives than with calcium-blocking agents, converting enzyme inhibitors or diuretics. Burchardt *et al*[8] reported a greater prevalence of cardiovascular complications in patients with hypertension and comorbid ED.

The development of the International Index of Erectile Function (IIEF), a validated, self-administered, five-item questionnaire to evaluate male sexual function,[17–20] and a widely used abbreviated version,[6,9–17] the IIEF-5, have facilitated the study of the prevalence of ED. Accordingly, the present study used the IIEF-5 to survey the prevalence of ED among hypertensive male patients attending internal medicine clinics at the Hammed General Hospital, HMC in Qatar. The aim of this study was to determine the prevalence of ED, its severity and other sexual function domains among hypertensive patients visiting outpatient clinics of the Hamad Medical Corporation.

## MATERIALS AND METHODS

This is a cross-sectional study aiming to investigate the prevalence of ED, its severity and other sexual function domains in hypertensive male patients attending internal medicine clinics at Hammed General Hospital, HMC in Qatar. The study was conducted between January and December 2006. Of the 850 hypertensive Qatari and nonQatari patients who were approached for the survey, 208 men were excluded from the study because either they declined to give their consent or they did not complete their questionnaires, resulting in 642 hypertensive patients (75.5%) being included in this study.

Hypertension was diagnosed when the average of two or more diastolic blood pressure measurements on at least two subsequent visits (at least two weeks apart) was 90 mmHg or when the average of multiple systolic blood pressure readings on two or more subsequent visits was consistently equal or greater than 140 mmHg.[3,4] All patients completed a detailed questionnaire addressing their general medical history, with special emphasis on hypertension history (i.e., duration of hypertension, type of antihypertensive treatment, compliance and presence of any complications). Sexual function was evaluated with the IIEF.[18–21]

Sexual function was assessed with the Arabic-translated version of IIEF.[18–21] Prior to the beginning of the study, the Arabic translation of IIEF was judged by 50 persons for clarity and conformity with the local culture and stated to be appropriate. Reliability analysis showed that the Cronbach's alpha value was 0.85 for the hypertensive group. The IIEF and its scoring system were found to be a reliable and valid measure of the five relevant domains of sexual function in men, including erectile functions (EF) orgasmic function (OF), sexual desire (SD), intercourse satisfaction (IS) and overall satisfaction (OS). The IIEF items EF, IS and OF are considered to predominantly reflect physical functions and SD and OS to mainly reflect psychological functions. The responses to each of the five questions were rated on a 0 to 5 points scale[17–20] and the total IIEF score ranged from 0-25, higher score indicating better sexual function. Direct help was given to the patients during completion of the IIEF if needed; using the IIEF scores, patients were classified as having no (22 to 25), mild (17 to 21), moderate (12 to 16) or severe (1 to 11) ED; a higher score indicates better function.[18–21]

Approval for the study was obtained from the medical ethics committee of the Hamad Medical Corporation. A consent form was obtained from the selected participants after the explanation of aims and the nature of the study. All interviews were conducted privately and confidentiality and anonymity of the participants was mainitained.

Student *t*-test was used to ascertain the significance of differences between mean values of two continuous variables and Mann-Whitney was used as non-parametric test. Chi-square and Fisher exact test were performed to test for differences in proportions of categorical variables between two or more groups. Pearson's correlation coefficient was used to evaluate the strength of concordance between variables. The level *P*<0.05 was considered as the cutoff value for significance.

## RESULTS

The mean ± SD age of all patients was 57.1 ± 11.9 years (range 25 to 75). The mean patient age at the detection of hypertension was 48 ± 9.1 years. Patients received therapy a mean of 7.4 ± 2.6 years after the diagnosis of hypertension and were treated for a mean of 12 ± 7.9 years for the disease. Only 18% sought medical treatment for ED.

Table 1 shows the socio-demographic and other characteristics of the studied participants and the percentage of sexual dysfunction in the hypertensive men. Of the 642 patients, 537 (83.6%) were married, 264 (41.1%) were Qatari nationals, 143 (22.3%) were currently smokers and 243 (37.8%) were obese. The prevalence of ED in hypertensive patients was 58.3%. Around 21.2% of hypertensive men reported severe ED, 20.7% moderate ED and 16.4% mild ED.

**Table 1:** The socio-demographic characteristics and prevalence of erectile dysfunction among hypertensive men studied

Variables	Hypertensive me (N =642) Frequency (n)	Percentages
Age group (years)		
<40	49	7.6
40-49	130	20.2
50-59	200	31.2
>60+	263	41.0
Body mass index		
Normal weight (<25 kg/m^2^)	192	(29.9)
Overweight (25-29 kg/m^2^)	207	(32.3)
Obese (≥ 30 kg/m2)	243	(37.8)
Natinality		
Qatari's	264	41.1
Non Qatari's	378	58.9
Marital status		
Single, divorced or widowed	105	16.4
Currently married	537	83.6
Educational level		
Illiterate	138	21.5
Primary	123	19.2
Intermediate	119	18.5
Secondary	157	24.5
University	105	16.4
Occupation		
Retired	123	19.2
Business	141	22.0
Clerical /administrative	223	34.7
Police/military	51	7.9
Professional	104	16.2
Smoking status		
Never smoked	300	46.7
Ex-smoker	199	31.0
Current smoker	143	22.3
Erectile dysfunction		
Yes (<21 IIEF)	374	58.3
No (22-25)	268	41.7
Severity of ED by IIEF score		
Severe (1-11)	136	21.2
Moderate (12-16)	133	20.7
Mild (17-21)	105	16.4
None (22-25)	268	41.7

IIEF = International index of erectile function

Table 2 provides the mean scores for the different domains of the IIEF for all 642 men. Patients with ED had significantly lower scores for the overall and individual items of the IIEF than those with no ED.

**Table 2:** Mean ± SD scores of all patients according to International index of erectile function domains

IIEF^1^ domain	Patients with ED^2^ N = 374	Patients with NO. ED^2^ N = 268	*P*-value
Erectile function	1.99 ± 0.70	4.26 ± 0.44	<0.001
Sexual desire	2.50 ± 0.88	4.50 ± 0.50	<0.001
Orgasmic function	2.66 ± 0.98	4.70 ± 0.46	<0.001
Intercourse satisfaction	2.97 ± 1.21	4.87 ± 0.33	<0.001
Overall satisfaction	3.13 ± 1.30	4.89 ± 0.35	<0.001
Overall score	2.71 ± 0.98	4.64 ± 0.20	<0.001

1 IIEF = International index of erectile function,

2 ED = Erectile dysfunction.

Table 3 lists the comorbidity with diseases and risk factors of all patients. These diseases/risk factors were significantly more prominent in the patients with ED than in those with normal ED. Likelihood Chi squared for association of ED with hypercholesterolemia was associated (χ^*2*^ = 7.10; *P*=0.009), similarly for diabetes (χ^*2*^ = 5.65; *P*=0.022), for hypertriglyceridemia (χ^*2*^ = 4.94; *P*<0.033) and myocardial ischemia (diagnosed from medical records or by ECG changes) (χ^*2*^ = 4.56, *P*=0.042).

**Table 3:** Erectile dysfunction risk factors in all patients

Comorbidity with a disease or a risk factor	Total (n=642) No. (%)	Normal erectile function (n=268) No. (%)	Erectile dysfunction (n=374) No. (%)	*P*-value
Hypercholesterolemia	159 (24.8)	52 (32.7)	107 (63.7)	0.009
Diabetes	299 (46.6)	110 (36.8)	189 (63.2)	0.022
Hypertriglyceridemia	158 (24.6)	54 (34.2)	104 (65.8)	0.033
Myocardial ischemia	126(19.6)	42 (33.3)	84 (66.7)	0.042
Currently smoking	143 (53.2)	68 (47.6)	75 (52.4)	NS

Table 4 presents the relationship between erectile function using IEEF and each group of antihypertensive drugs. A highly statistically significant association was found between the type of antihypertensive treatment taken and ED. Those on beta blockers (BB) were more likely to have ED than other antihypertensive medications.

**Table 4:** Relationship between erectile function using International index of erectile function and each group of antihypertensive drugs

Antihypertensive group	Total (n=642) No. (%)	Normal erectile function (n) No. (%)	Erectile dysfunction (n) No. (%)	*P*-value
ACE inhibitors	426 (66)	164 (38.5)	262 (61.5)	0.024
Diuretic	261 (40.7)	95 (36.4)	166 (63.6)	0.0282
Beta blocker	153 (24)	46 (30.1)	107 (69.9)	0.001
Calcium channel blocker	138 (18)	43 (31.2)	95 (68.8)	0.006

Furthermore, the Pearson's correlation between the duration of hypertension and the duration of weak erections (erectile function item of the IIEF) (r = 0.63, *P*<0.01) was highly statistically significant.

## DISCUSSION

Although hypertension is considered a disease with few subjective symptoms,[11] the rate of ED among hypertensives (58.3%) was significantly higher than its equivelant among the general population[6] as well as that reported among hypertensive men in other Arab countries as Egypt (43.2%).[10] Our study showed that 41.9% of our patients had either moderate or severe ED compared with 38.2% reported by Mittawae *et al*.[10] The difference in prevalence could be partially explained by the percentage of those sexually active in the two samples of Qatar and Egypt. Only 21.2% of our patients were sexually inactive compared with 30% reported in Egypt.[22] Our finding was also near to another recent multicenter Spanish study reporting a prevalence of 45.8% of ED in 2130 patients with hypertension.[15] The Massachusetts male aging study reported only 11% of patients with severe ED[6] which is obviously less than our results. Moreover, other studies have reported different or less prevalence rates of ED in men with hypertension.[6–14] These controversies might be related to the nature of the population sample examined, the sample size, the percentage of sexually inactive, the mode of treatment of hypertension, the medication doses and combinations of therapy, the level of hypertension control, patients’ compliance to treatment, different countries’ lifestyle and the type of instrument used to assess erectile function.[20–21] However, although differences exist among prevalence rates of ED in hypertension, all the studies showed a greater prevalence of ED in patients with hypertension than in the normal population. Decreased periphral circulation along with essential hypertension may be related to erectile dysfunction.[8] Also, side-effects of hypertensive drugs sometimes result in erectile dysfunction.[9,23]

In Greece[14] ED was evaluated with the same tool and it was found in 35.2% of patients with essential hypertension compared with 14.1% of normotensive subjects. Patients with essential hypertension had more severe ED than their normotensive counterparts. This is confirmative with the current study outcome.

A variety of physical and psychological factors are involved in erectile function and the alteration of one or more factors may lead to ED.[10] Diabetes,[3,6,10,12,23] hyperlipidemia,[6,10,12,23] hypercholesterolemia[1,6,10] and smoking[19] are all well-known risk factors of cardiovascular disease and ED. This is consistent with the present study results. The significantly greater occurence of these risk factors in our patients with both hypertension and ED compared with the likelihood in patients with hypertension alone underscores the synergistic detrimental effects of these risk factors on erectile function. Owing to religious beliefs, alcohol consumption, a well-known risk factor of hypertension[20] was limited in our patients.

Most of our patients were being treated for hypertension since a long duration with a mean and SD of 12 ± 7.9 years. Generally, up to 25% of ED cases were related to medication side-effects and antihypertensive drugs were the most implicated class.[23] Many antihypertensive drugs may cause or exacerbate ED as a side-effect.[10,14,21–25] Several lines of evidence suggested that BB were associated with an increased risk of ED.[10,13,14,23–25] Similarly, our study showed that those on BB were more likely to have ED than those on other antihypertensive medications. However, the exact mechanism of ED caused by BB remained unclarified. Its adverse effect on sexual activity may be due to an interference with the adrenergic system's function involved in the integration phase of erection and ejaculation.[14] ED is mostly readily reversible when the responsible drug is stopped or suitable alternative is given.[23]

Even though most of our patients regularly visited their physicians, only 18% sought medical treatment for ED. We speculated that most patients with hypertension were more concerned about their high blood pressure level and its life-threatening conditions than about their erectile status. This suggests increased patient awareness about the medical treatment for ED.

To the best of our knowledge, the current study is one of the few to investigate ED in patients with hypertension using a well-validated erectile function questionnaire all over the world[18–21] and the first in the GCC countries. However, the study still had its limitations. The first limitation was the level of hypertension control, which is a strong determinant of ED and was not accounted for in the current study. The second is measuring patients’ compliance to treatment. However, both, level of control and patients’ compliance were difficult to be studied in the context of our limited logistics. Moreover, only 20-30% of hypertensive patients are under sufficient blood pressure control,[25] for which low compliance to antihypertensive treatment[26] is considered to be one of the main reasons. Ironically, adherence to antihypertensive treatment might be difficult in case ED arises during its course and affects the quality of patient's life.[11] Because ED related to antihypertensive medication was proved to be a dose-related problem and probably not all BB have the same negative effects on erectile function.[14]

## CONCLUSION

Our results have shown a greater prevalence of ED in the hypertensive men in the population of Qatar. The presence of another risk factor in addition to elevated blood pressure can increase the likelihood of ED.
